# Differential Axonal Conduction Patterns of Mechano-Sensitive and Mechano-Insensitive Nociceptors – A Combined Experimental and Modelling Study

**DOI:** 10.1371/journal.pone.0103556

**Published:** 2014-08-19

**Authors:** Marcus E. Petersson, Otilia Obreja, Angelika Lampert, Richard W. Carr, Martin Schmelz, Erik Fransén

**Affiliations:** 1 School of Computer Science and Communication, KTH Royal Institute of Technology, Stockholm, Sweden; Stockholm Brain Institute, KTH Royal Institute of Technology, Stockholm, Sweden; 2 Dept. of Anaesthesiology, Universitaetsmedizin Mannheim, Univ. of Heidelberg, Mannheim, Germany; 3 Institute of Physiology and Pathophysiology, Friedrich-Alexander-Universität Erlangen-Nürnberg, Erlangen, Germany; The Hebrew University Medical School, Israel

## Abstract

Cutaneous pain sensations are mediated largely by C-nociceptors consisting of both mechano-sensitive (CM) and mechano-insensitive (CMi) fibres that can be distinguished from one another according to their characteristic axonal properties. In healthy skin and relative to CMi fibres, CM fibres show a higher initial conduction velocity, less activity-dependent conduction velocity slowing, and less prominent post-spike supernormality. However, after sensitization with nerve growth factor, the electrical signature of CMi fibres changes towards a profile similar to that of CM fibres. Here we take a combined experimental and modelling approach to examine the molecular basis of such alterations to the excitation thresholds. Changes in electrical activation thresholds and activity-dependent slowing were examined *in vivo* using single-fibre recordings of CM and CMi fibres in domestic pigs following NGF application. Using computational modelling, we investigated which axonal mechanisms contribute most to the electrophysiological differences between the fibre classes. Simulations of axonal conduction suggest that the differences between CMi and CM fibres are strongly influenced by the densities of the delayed rectifier potassium channel (K_dr_), the voltage-gated sodium channels Na_V_1.7 and Na_V_1.8, and the Na^+^/K^+^-ATPase. Specifically, the CM fibre profile required less K_dr_ and Na_V_1.8 in combination with more Na_V_1.7 and Na^+^/K^+^-ATPase. The difference between CM and CMi fibres is thus likely to reflect a relative rather than an absolute difference in protein expression. In support of this, it was possible to replicate the experimental reduction of the ADS pattern of CMi nociceptors towards a CM-like pattern following intradermal injection of nerve growth factor by decreasing the contribution of K_dr_ (by 50%), increasing the Na^+^/K^+^-ATPase (by 10%), and reducing the branch length from 2 cm to 1 cm. The findings highlight key molecules that potentially contribute to the NGF-induced switch in nociceptors phenotype, in particular Na_V_1.7 which has already been identified clinically as a principal contributor to chronic pain states such as inherited erythromelalgia.

## Introduction

The peripheral mechanisms underlying chronic pain states are not completely understood. Most nociceptors innervating human skin are unmyelinated and slowly conducting. Among C-nociceptors there are both mechano-sensitive (CM) and mechano-insensitive (CMi) fibres and while both classes are involved in mediating inflammatory hyperalgesia, CMi fibres, are particularly important for mechanical hyperalgesia [1], [2], axon reflex erythema [3], central sensitization [4] and spontaneous activity in chronic pain patients [5]. It is therefore important to understand the molecular basis for the functional differences distinguishing these two nociceptor types in humans [6]. In addition to their different functional roles and their sensory responsiveness, CM and CMi fibres exhibit distinct differences in their axonal characteristics which differentiate between the two classes virtually without overlap. Specifically, CMi nociceptors display characteristically more activity-dependent slowing (ADS) of conduction velocity (CV) when stimulated at relatively low (0.125 to 2 Hz) frequencies and more supernormality when probed by double pulses at short intervals (i.e., 40–250 ms) [7]–[9]. Finally, the innervation territories of human CMi fibres are larger than those of their CM counterparts presumably corresponding to longer terminal branches.

While in normal subjects CM and CMi nociceptors can be readily differentiated according to their sensory and axonal characteristics, these differences can change under pathophysiological conditions [10]. In chronic pain patients, ADS in both CM and CMi fibres was decreased [11], [12] and it is tempting to suggest that such changes in axonal excitability could contribute to chronic pain. Thus, while basic functional differences between the two nociceptor classes certainly exist, the molecular entities and structural factors that underlie these differences are not known. Recent experimental evidence indicates that it is possible to selectively modulate ADS in cutaneous CMi fibres in the pig [13]. One week after intradermal injection of nerve growth factor into pig skin, ADS patterns of CMi resembled that of CM nociceptors although they remained insensitive to mechanical stimuli [13]. This result indicates that, independent of mechanical sensitivity, it is possible to change the axonal signature from a CMi to a CM pattern. The key question would subsequently be to identify the molecular entities that potentially underlie this change in ADS phenotype of the nociceptor classes. To examine this, a computational model [14] was used to replicate the axonal conduction properties in CM and CMi fibres from human and pig obtained from *in vivo* experiments. As candidates, we included resting membrane potential, K_dr_, K_M_, K_Na_, I_h_, Na_v_1.7, Na_v_1.8 as well as the Na^+^/K^+^-ATPase pump. Our key aim is to assess the axonal properties of nociceptors in-silico such that the above targets could be prioritized with respect to their likely contribution to the basic differences between CM and CMi nociceptors. This approach also provides hypotheses on the mechanism by which NGF can modify the axonal excitability of nociceptors.

## Methods

### Computational model

Simulations were performed using our computational CMi axon model [14]. Briefly, we developed a biophysically realistic multi-compartment model of an unmyelinated axon including several Hodgkin-Huxley-type ion channels (Na_V_1.7, Na_V_1.8, Na_V_1.9, K_dr_, K_A_, K_M_, K_Na_ and HCN), a Na^+^/K^+^-ATPase pump and intra- and extracellular Na^+^ and K^+^ concentrations. Structural aspects of the axon were also included in the model. A thin (0.25 µm) superficial axonal branch was modelled at skin temperature (32 degrees) which was connected via a conic segment to a thicker (1 µm) axon at normal body temperature (37°C) representative of a deep parent axon.

We focussed our modelling approach on those axonal characteristics that have been shown experimentally to separate CM and CMi fibres:

ADS induced by continuous stimulation at 2 Hz for 3 min is much more prominent in CMi fibres (human: 36.7%; pig: 30.1%) compared to CM fibres (human: 22.5%; pig: 14.3% [15]) and the two fibre types can be distinguished based on the amount of ADS observed at 2 Hz [16].
Although the likelihood of propagation failures increases in all peripheral C-fibres in an activity-dependent manner [17]failures are less prominent in CM fibres than in CMi fibres [18].
The resting CV of CM axons is significantly higher than of CMi fibres in both human (range 25–67%) and pig (44%) nociceptors, although there is some degree of overlap in the two populations [15], [16].
In the 100 ms period immediately following an action potential, CM fibres display less prominent super-normal conduction than CMi fibres [7], [8].
The innervation territory of human cutaneous CMi fibres is larger than that of CM fibres [19]and presumably reflects longer terminal branches.
The transcutaneous electrical threshold of CM fibres is much lower (4 mA) than that of CMi fibres (60 mA), as shown by Weidner et al. [16].

### NGF intradermal injections and extracellular single-fibre recordings in anesthetized pigs, *in vivo*

Animal experiments were approved by the regional council in Baden-Wuerttemberg and by the ethics committee of the University of Heidelberg, Germany (file number: Az 35-9185.81/G-145/08). Nerve growth factor (NGF, Sigma-Aldrich, Deisenhofen, Germany) was injected intradermally into the skin of the inner hind limb in 21 domestic pigs (Sus scrofa domesticus; median bodyweight: 31.9 kg; age: 12±4 weeks; see [13]). Sedated animals [2 mg/kg azaperone (Stresnil, Janssen Pharmaceutica, Beerse, Belgium) and 1 mg/kg midazolam (Dormicum, Roche, Basel, Switzerland)] received either 2 or 8 µg NGF, dissolved into a volume of 200 µl and administered as 5 injections per limb.

**Figure 1:**
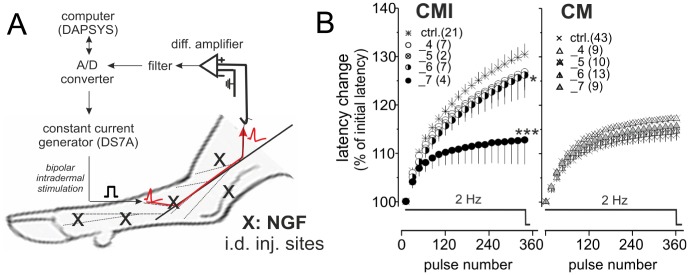
NGF reduces activity-dependent slowing of conduction in the mechano-insensitive fibres in a time-dependent manner. (**A**) Experimental set-up. Schematic representation of the setup for electrophysiological recordings in the pig skin, *in vivo*. Close-up of the dissected saphenous nerve, placed in a groove on a metal mirror. A pair of microelectrodes is used for intradermal stimulation in the innervation territory of the saphenous nerve. The nerve endings are stimulated with repetitive rectangular pulses (0.5 ms duration; 0.25 Hz frequency). NGF was injected intradermally in the saphenous nerve innervation territory (5 injections per hind limb, 20 µl per injection site; total dose: 2 or 8 µg), 4 to 7 days before recording day. (**B**) ADS patterns during stimulation at 2 Hz for 3 min are shown for mechano-insensitive afferents (*CMi*; circles, left panel) and for mechano-sensitive afferents (*CM*; triangles, right panel) in control situation (*ctrl.*) or at 4 to 7 days after NGF treatment (days indicated by numeric value after “_”. Number of units is given in parentheses. Changes are shown relative to the initial latency recorded after pause. Statistical differences between NGF-treated versus control units (ANOVA, repetitive measurements with LSD post hoc test) are marked by asterisks (*).

Extracellular recordings from the saphenous nerve were performed four to seven days following NGF administration (figure 1), according to the experimental protocol described previously [13]. General anesthesia was induced with Propofol (Fresenius, Bad Homburg, Germany) 2 mg/kg i.v. and maintained with pentobarbital (Narcoren, Merial, Halbergmoos, Germany) 8–18 mg/kg/hour. Vital parameters were monitored continuously throughout the procedure. Rocuronium (Esmeron, Organon International, BH OSS, Netherlands) 0.5 mg/kg and succinylcholine (Lysthenon, Nycomed, Unterschleissheim, Germany) 7–8 mg/kg/h were administered for muscle relaxation. Saphenous nerves were exposed over a length of about 5 cm proximal to the knee. The “teased fibre” technique was employed to record action potentials extracellularly in response to constant current electrical stimulation (20 mA; 0.5 ms; DS7A, Digitimer Ltd., Hertfordshire, UK) within the cutaneous receptive field. Electrical stimuli were applied at 0.25 Hz via intradermal non-insulated microneurography electrodes (FHC Inc., Bowdoin, ME, USA) (Fig. 1A). The distance from the most proximal stimulation needle in the skin and the recording electrode was used to estimate CV. When determined immediately after a 2-min pause all fibres in this study had CV values ≤2 m/s. Extracellular signals were amplified (Low-Noise Voltage Preamplifier Model 5113, Ametek Inc., TN, USA), filtered (bandwidth 100–3000 Hz; Model 3364, Krohn-Hite Corp., Brockton, MA, USA), audio monitored and digitized at a sampling rate of 32 kHz using DAPSYS 7.0, a joint hardware and software system designed for real-time acquisition, window discrimination and latency measurements of action potentials [15].

During ongoing intradermal electrical stimulation (0.25 Hz), mechanical stimuli (150 mN; Semmes-Weinstein calibrated monofilaments) were applied to the skin to localize the peripheral receptive field of the unit under study. At mechano-sensitive spots, action potentials induced by mechanical stimulation led to “marking” of the electrically-evoked action potential, i.e. an increase in latency [20], [21]. Fibres displaying marking upon stimulation with 150 mN were subsequently screened for sensitivity to innocuous stimulation with a paintbrush and if unresponsive, were classified as mechano-sensitive nociceptors (CM [15]). Receptive fields of mechano-insensitive nociceptors (CMi) were determined using electrical stimulation and collision techniques [22].

ADS was measured as the total latency change during continuous stimulation at 2 Hz for 3 min (Fig. 1B). For electrical stimulation a pulse width of 0.5 ms was used and the stimulation intensity was set to twice the electrical threshold determined by intracutaneous stimulation at 0.25 Hz [13].

### Stimulation protocols *in silico*

**Figure 2:**
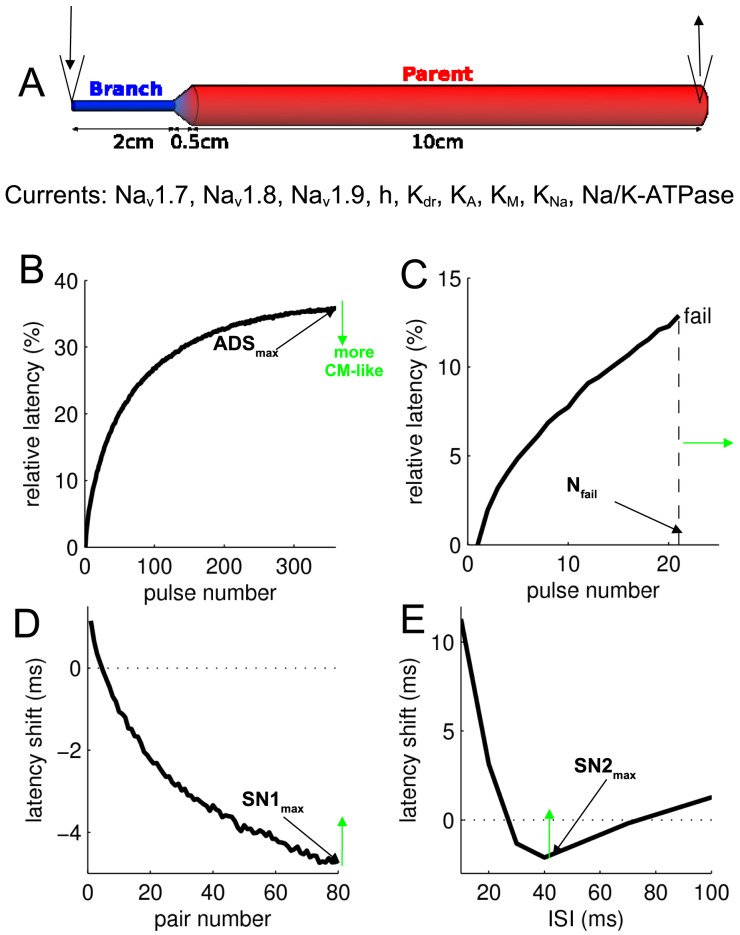
Functional properties of default CMi model. (**A**) Axon model geometry. A branch of length (L) 2 cm and diameter (D) 0.25 µm is connected to a parent axon (L: 10 cm; D: 1 µm) via a cone (L: 0.5 cm, linearly increasing diameter from 0.25 to 1 µm) (**B–E**) Protocols used to validate the models. Arrows indicate how results should change for the model to represent a CM rather than a CMi fibre. (**B**) Activity-dependent slowing (ADS). Stimulation with 360 pulses at 2 Hz results in maximal slowing ADS**_max_**. (**C**) Activity-dependent conduction failure. 2 Hz with 1.2× threshold current. N_fail_ is the pulse number for which failures first occur. (**D**) Supernormality (SN) induced by 80 pulse pairs (ISI = 50 ms) given at 1 Hz. SN1_max_ is defined as the latency shift of the 80th pulse pair. (**E**) Recovery-cycle protocol with background frequency 0.5 Hz. 100 pre-pulses (not shown) followed by pulse pairs of varying inter-stimulus intervals (ISI). Maximal supernormality (SN2_max_) is measured at the maximal negative latency shift.

Geometry of the peripheral neuron and currents included in the model are summarized in Fig. 2A. Stimulation protocols used in this study are shown in Figure 2 (B–E). ADS was measured in a manner similar to that used *in vivo*, namely by applying repetitive stimulation pulses at 2 Hz (5 ms, 0.1 nA) for 3 min. The maximal ADS obtained was denoted ADS_max_ (Fig. 2B). The tendency toward activity-dependent conduction failures was measured *in silico* by counting the number of action potentials conducted without failure when the stimulation current amplitude was set to 20% above threshold (N_fail_ is the pulse number for the first failure to occur; Fig. 2C). Supernormal conduction (shifts of responses to shorter latencies) is measured *in vivo* by two protocols [7]–[9], which use either fixed [7] or varying [8] inter-stimulus intervals (ISI),. Accordingly, supernormality was assessed *in silico* using both protocols. In the first protocol (SN1) we repetitively applied pulse pairs with a fixed ISI of 50 ms and at a repetition rate of 1 Hz (resulting in maximal supernormality SN1_max_ shown in Fig. 2D). In the second protocol (SN2) we first induced ADS slowing (again, in accordance with *in vivo* experiments [7]) by initially applying 100 single pulses at 0.5 Hz, and then using pulse pairs with varying ISIs (3, 5, 10, 20, 30, 40, 50, 70, 100, 150, 200, 250, 500 ms; repetition rate of pulse pairs was 0.5 Hz). Maximal supernormality SN2_max_ was calculated as the maximal negative latency shift, as indicated in the figure (2E). Figure 2 also indicates the direction (green arrows) in which the curves should change in order to make a CMi fibre behave more like a CM fibre.

### Simulations and score system

**Figure 3:**
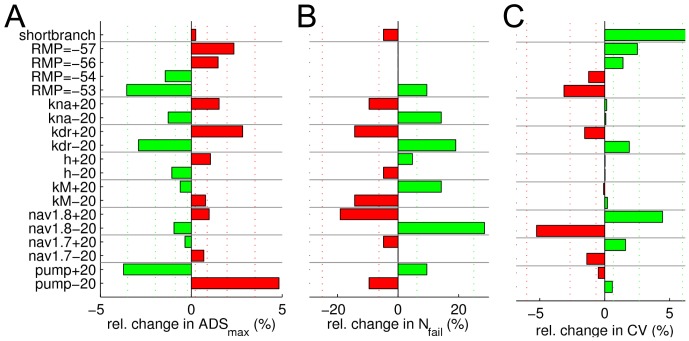
Effect of changing ion channel and pump densities, resting membrane potential and branch length on slowing, conduction failure and initial conduction velocity. Bars indicate that a parameter change led to a model function more (green), respectively less (red) like a CM fibre. See text for details on the vertical dotted score lines. (**A**) Activity-dependent slowing protocol. Bars calculated as ADS**_max_**/ADS**_max,DEF_**, where ADS**_max,DEF_** is ADS**_max_** in the default model. (**B**) Activity-dependent conduction failure protocol. Bars calculated as N**_fail_**/N**_fail,DEF_**, where N**_fail,DEF_** is N**_fail_** in the default model. (**C**) Initial conduction velocity (CV). Bars calculated as CV/CV**_DEF_**, where CV**_DEF_** is CV in the default model.

**Figure 4:**
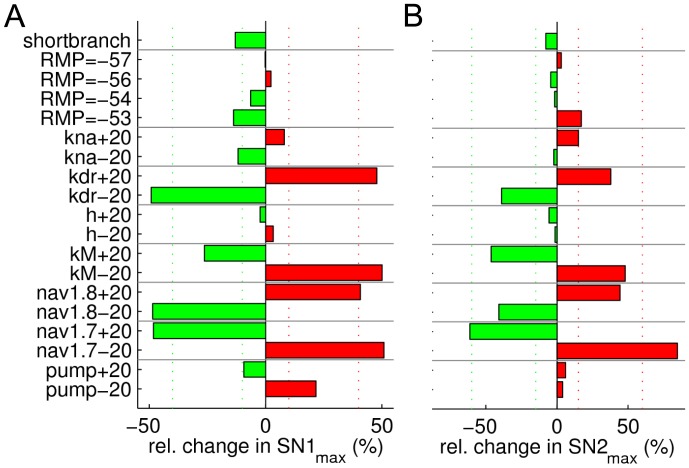
Effect of changing ion channel and pump densities, resting membrane potential and branch length on supernormal conduction. Same colour coding as in fig. 3. See text for details on the vertical dotted score lines. Supernormality protocols were used for calculating SN1_max_ (**A**) and SN2_max_ (**B**). Bars calculated as (SN1_max_/IL)/(SN1_max,DEF_/IL_DEF_) (**A**) and (SN2_max_/IL)/(SN2_max,DEF_/IL_DEF_) (**B**), where IL denotes the initial latency.

To examine whether differences in the conductive properties of CM and CMi fibres can be attributed to differences in resting membrane potential (RMP) and axonal ion channel and pump densities, values of RMP (±1 and ±2 mV from default RMP = −55 mV) and the density of each channel or pump were systematically varied (by ±20%). Figures 3 and 4 show how varying these parameters affects ADS, the likelihood of conduction block, axonal resting CV and supernormality. Notably, the effects of varying because, Due to their small maximal conductance values in our model, these channels only marginally influence the parameters investigated and results for K_A_ and Na_V_1.9 have been excluded from the tables. In addition, to examine the role of the length of the nerve terminal branch, a region in which ADS is suggested to be prominent [9], [23]), the branch length was varied from 2 to 1 cm.

The deviations caused by the parameter changes are shown in Figures 3 and 4 as green or red bars. Green bars indicate parameter changes that modified the behaviour of the default CMi fibre model such that it showed features more like a CM fibre, while red bars indicate the opposite (model responds less like a CM fibre). Deviations were scored on an exponentially increasing scale as indicated by the vertical dotted lines in Figures 3 and 4. Each response bar (green; red) exceeding the threshold score (Fig. 3 and 4, vertical lines) yielded a positive or negative point, respectively. Score lines were placed as follows: ±A· (j/N)^2^, with index j = 1,2,…,N (For ADS_max_: A = 3.5, N = 4; For N_fail_: A = 25, N = 2; For CV: A = 6, N = 3; For SN1_max_: A = 40, N = 2; For SN1_max_: A = 60, N = 2), where the values of A were chosen according to the magnitude of protocol-specific changes. Specifically, A was chosen to be slightly smaller than the maximal difference evoked by the parameter changes. N indicates the number of score lines and was chosen for each measure and corresponds to how confident we are that results from CM and CMi fibres differ significantly. Specifically, N is high for slowing (ADS_max_: N = 4) and supernormality (SN1_max_+SN2_max_ = 2+2) since there is quantitative human *in vivo* data for these measures, and the overlap of CM and CMi fibres is small [16]. N is smaller for the CV (N = 3) because there is more overlap in CV values of CM and CMi [16]). Finally, N is even smaller for the propagation-failure tendency measure (N_fail_: N = 2) because the difference in propagation-failure tendency in human CM and CMi fibres has not been specifically addressed *in vivo*, although indirect supporting evidence from a porcine study [18], and a study on human fibres shows that a high failure tendency is associated with a large amount of ADS [24].

## Results

Application of NGF has been shown to alter axonal activity-dependent slowing of conduction, ADS, of CMi fibres [13]. To test the hypothesis that NGF-induced changes in densities of ion channels and pumps could underlie the changes in ADS, we first re-analyzed our experimental data involving NGF application (see Fig. 1B) and studied the changes according to days after NGF-application. To enable comparisons of excitability differences between fibres as well as between experimental data and model output, we also analysed experimental data on electrical thresholds for CM and CMi fibres. In our computational study, we started from the model which was found to adequately replicate features of axonal conduction in a CMi axon; action potential threshold, conduction velocity (CV), ADS and recovery cycles [14]. Using this model, we studied to what extent changes to ion channel and pump densities would be associated with shifts in fibre properties from CMi to CM-like.

### NGF reduced ADS in porcine CMi nociceptors in a time-dependent manner

In CMi nociceptors and mechano-sensitive CM fibres from pig saphenous nerves, changes in latency upon continuous stimulation at 2 Hz for 3 min were recorded *in vivo* following NGF application (see Fig. 1B). NGF treatment reduced the amount of ADS in CMi fibres (Fig. 1B, left panel). In contrast, no alteration in the pattern or total ADS of CM fibres was observed (Fig. 1B, right panel). The decrease in slowing in CMi units became progressively larger over time, such that seven days after NGF administration, the slowing pattern in the CMi and CM fibres was almost identical. This finding is based on previously published data [13] and has been re-analysed in order to illustrate the time-dependent effect of NGF. Thus, the time course of ADS changes parallels that of NGF-induced protein expression pattern alterations [25], thereby supporting the link between NGF application and functional expression changes of axonal ion channels and pumps.

### ADS depends on resting membrane potential, K_dr_ and Na^+^/K^+^-ATPase

In our model, we first explored how changes in ion channel and pump densities, resting membrane potential (RMP) and branch length affected ADS (Figure 3A, green bars indicate a trend in the direction towards CM fibre properties). Three parameter changes – depolarizing resting membrane potential, reducing K_dr_ and increasing Na+/K+-ATPase – rendered ADS properties more CM-like.

### CV and propagation failure

We subsequently analysed influences on propagation failure and velocity of propagation in the model. The simulation results shown in Figure 3B indicate that the risk of propagation failures is particularly strongly affected by changes of Na_V_1.8 density. A 20% reduction of Na_V_1.8 increases the risk of propagation failure by more than 20%. Furthermore, simulation results indicate that, CM fibres may be equipped with more Na_V_1.7 and Na_V_1.8, less K_dr_ conductance, and a hyperpolarized resting membrane potential, in order to achieve their faster CV compared to CMi fibres (Figure 3C). We also see that a reduction in branch length from 2 to 1 cm causes an increase in CV by 6.6%.

### Electrical thresholds, experiment and simulation

In human, transcutaneous electrical thresholds of CM fibres are much lower (4 mA) than in CMi fibres (60 mA) [16]. Consistent with this, we find in our experiments that transcutaneous stimulation of porcine CMi fibres gave a rheobase of 36.8 mA (n = 2), 30-times larger than the median value of 1.4 mA (n = 9) in CM nociceptors. In contrast, CMi and CM nociceptors have similar rheobase when stimulated intracutaneously (median of 1.69 mA (n = 6 CMi) versus 0.71 mA (n = 14 CM), p>0.1; U-test). Such *in vivo* differences upon transcutaneous (but not intracutaneous) stimulation might derive from the fibre's location in the skin. Action potential initiation in deeper fibres may need higher stimulation intensity than in those situated more superficially. We also performed simulations to measure the influence of ion channel and pump densities, resting membrane potential and branch length on current threshold in the model. Currents were injected via an intracellular electrode (see Methods). Consistent with the lack of variance in current threshold for intracutaneous stimulation, all resulting changes in Ithres were <3%, thereby not statistically significant (data not shown).

### Supernormal conduction depends on Na_V_1.7, Na_V_1.8, K_dr_ and K_M_

To further study the activity-dependent properties of CM and CMi fibres *in silico*, we analysed differences in excitability during recovery cycles. In our model, two stimulation protocols were used to assess changes in super-normal conduction, SN1 and SN2 (see Methods). Figure 4 displays the results obtained during stimulation using a fixed (SN1; Fig. 4A) respectively varying (SN2; Fig. 4B) inter-stimulus interval. The results obtained with these two protocols suggest that CM fibres might have more Na_V_1.7 and K_M_, and less Na_V_1.8 and K_dr_ conductance.

### Predicted differences between CM and CMi fibres

**Figure 5:**
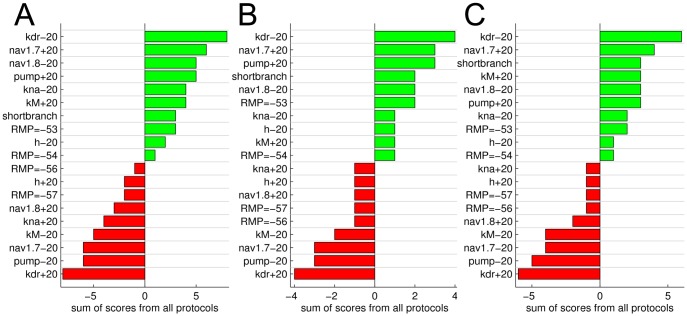
Results from all stimulation protocols weighed together by use of the scoring system. Large bars should be interpreted as strong candidates for explaining what change makes a CMi fibre more CM-like (green) or unlike (red). The results suggest that, compared to CMi fibres, CM fibres have less K_dr_ channels, more Na_V_1.7 channels, etc. (**A**): Exponentially increasing score lines (as in Figures 3 and 4): ±A·(j/N)^2^, with j = 1,2,…,N. For ADS_max_: A = 3.5, N = 4; For N_fail_: A = 25, N = 2; For CV: A = 6, N = 3; For SN1_max_: A = 40, N = 2; For SN1_max_: A = 60, N = 2. (**B**): Like (A) but with fewer exponentially separated score lines: For ADS_max_: N = 3; For N_fail_: N = 1; For CV: N = 2; For SN1_max_: N = 1; For SN1_max_: N = 1. (**C**): Similar to (A) but with linearly separated score lines: ADS_max_: ±1,2,3,4; N_fail_: ±10,20; CV: ±2,4,6; SN1_max_: ±20,40; SN2_max_: ±30,60.

Our simulations revealed that over all tested protocols the main contributing factors are similar. For example, increased Na_V_1.7 conductance leads to less ADS, less supernormality and faster CV, all characteristics of CM nociceptors (see Figures 3, 4). However, opposing results between protocols were observed as well, e.g. decreased Na_V_1.8 and depolarized resting membrane potential leading to reduced ADS (becoming more CM-like) but with slower CV (becoming less CM-like). In order to combine and compare the results from all protocols, we calculated the sum of evaluation scores from all protocols by using the score scheme indicated in Figures 3 and 4. The results are summarized in Figure 5A. Changing score schemes did not lead to major changes of the results, as shown in Figure 5B and C. Among K^+^ channels, K_dr_ stands out by having an overall strong influence on all propagation properties tested, and results strongly indicate that the axonal K_dr_ density is lower in CM than in CMi fibres. Furthermore, our results indicate that CM fibres have less K_Na_ and more K_M_, although it should be noted that these channels had less influence than K_dr_. For the two Na^+^ channels Na_v_1.7 and Na_v_1.8, it is interesting to note that in principle their individual influences oppose one another. The model strongly suggests that CM fibres have more Na_V_1.7, but less Na_V_1.8, compared to CMi fibres. The model further suggests that the expression, or perhaps the electrogenic effect, of the Na+/K+-ATPase is relatively large in CM fibres. The hyperpolarization-activated h-current, on the other hand, might have a low conductance in CM but is relatively higher in CMi nociceptors. The influence of RMP and branch axon length is not straightforward, see also Discussion.

### Combined parameter changes can modify CMi fibre to represent CM fibre

**Figure 6:**
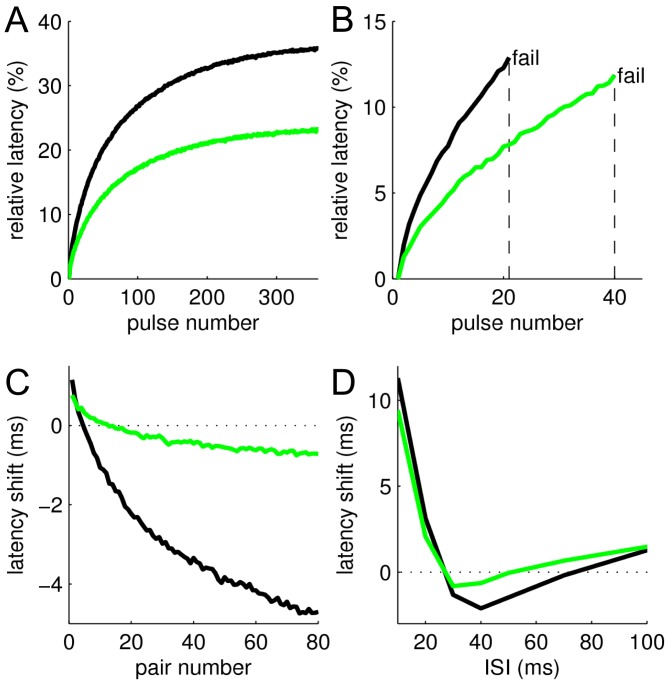
Example simulations with one selected parameter change modifying the CMi model (black, default values) to display CM fibre like function (green). Compared to the default CMi model (black), the CM-like model (green) has a shorter branch length (1 rather than 2 cm), 50% lower K_dr_ channel density and 10% higher pump density. Figures show how (**A**) ADS, (**B**) conduction failures, (**C**) SN1 and (**D**) SN2 were affected by these changes.

From the results above it is clear that more than one parameter is involved in creating the observed differences between CM and CMi fibres and we could identify several promising candidates. It is also clear that some parameter alteration produces changes in opposite directions depending on the applied protocol, as discussed above. We were therefore interested to see if we can identify one set of parameter changes that would turn a CM into a CMi fibre. In Figure 6 we illustrate that a small set of parameter changes can not only modify the CMi model to be more similar to a CM model (in relative terms), but also quantitatively modify the model to represent an CM fibre in absolute terms. The figure shows simulation results from both the default CMi model (black) and from a CM model (green) in which selectively the following parameters were altered: the branch length was decreased from 2 cm to 1 cm, K_dr_ channel density was reduced by 50%, and pump density was increased by 10%. This set of changes increases the initial CV by 12% (from 0.59 to 0.66 m/s, not shown), decreases ADS_max_ from 36% to 23% (Fig. 6A), increases N_fail_ from 21 to 40 (Fig. 6B), reduces SN1_max_ from 4.7 to 0.7 ms (Fig. 6C) and reduces SN2_max_ from 2.1 to 0.8 ms (Fig. 6D). We further note that an assimilation of CM and CMi fibre properties could be obtained with modest changes of parameter values.

### Correlation of fibre properties across protocols

**Figure 7:**
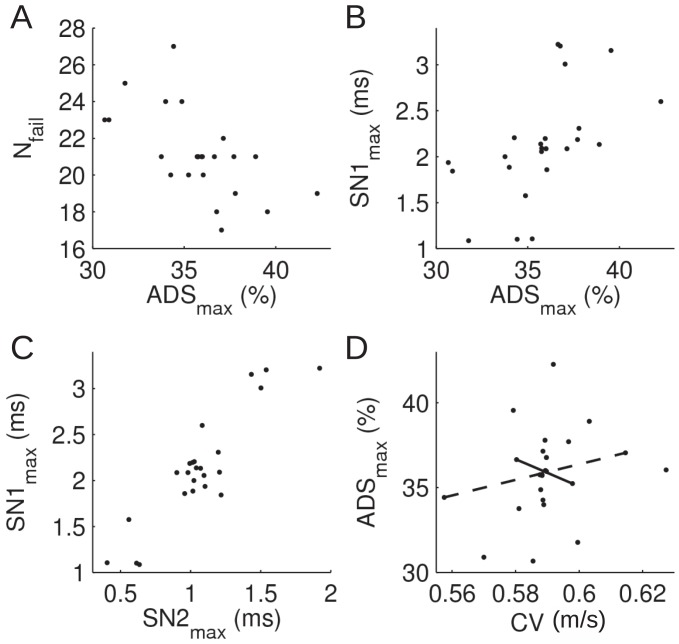
Correlation of data from Figures 3 and 4 across stimulation protocols. (**A–D**) Correlations between pairs of functional properties. In panel (**D**), the lines connect data points from models where the channel densities of Na_V_1.7 (solid, negative correlation) and Na_V_1.8 (dashed, positive correlation) were modified.

To refine our analysis of simulation and experimental data we compare results across all tested protocols (Figure 7). Axons exhibiting strong ADS typically have a tendency towards conduction failures [18] and this trend was also observed for the model axon (Figure 7A). Prominent ADS is associated with a marked supernormal conduction phase [7], [9], which we were able to reproduce in the simulated axon (Figure 7B). The strong correlation seen for the two protocols assessing supernormality in the model axon (SN1 and SN2; Figure 6C) is consistent with published experimental work [7], [9]. Simulation results indicated no correlation between initial CV and ADS (Figure 7D).

## Discussion

Based on the results presented here, we propose that variations in a certain set of ion channel and pump densities can explain many of the differences in axonal propagation properties observed experimentally between CM and CMi unmyelinated peripheral nociceptors. Specifically, our simulation results strongly suggest that, compared to CMi fibres, CM fibres are likely to have less K_dr_, but more Na_V_1.7 and Na^+^/K^+^-ATPase density. CM fibres may express less Na_V_1.8 and K_Na_ than CMi fibres but more K_M_. Possibly CMi contain less HCN and display a more depolarized resting membrane potential than CMi fibres. According to our simulations the length difference in CM and CMi innervation territories (branch lengths) explains some, but certainly not all, of the differences in axonal propagation properties (Figures 3 and 4). We also find that changes of modest magnitude (up to 50%) in only a small number of parameters (3) were sufficient to alter the axonal conduction properties of a CMi type fibre to a reasonable approximation of a CM fibre.

### Parameters with greatest influence on ADS, CV and supernormality

One aim of this study was to find candidate mechanisms that distinguish CM from CMi fibres. A clear benefit of using computational modelling is that all parameters are observable and can be manipulated in a controlled way. We can therefore make statements about how specific parameter changes affect axonal propagation properties. With regard to the differential properties of CMi and CM fibres, a few observations are noteworthy. Based upon simulations of ADS in C-fibres, we have previously postulated that intracellular Na^+^ accumulation is a potentially important contributing factor [14]. Based on this postulate, an increased pump density is consistent with reduced ADS, since more Na^+^ will be pumped out. It is less intuitive however, that a reduction in K_dr_ results in a similar effect. Possibly, less K_dr_ current leads to a slower repolarization after the action potential peak, which allows for a more complete inactivation of Na_V_1.8, and thereby less Na_V_1.8 current and consequently less Na-inflow during the next action potential, and thereby less ADS. Note that when compared to Na_V_1.7, Na_V_1.8 lasts longer during the action potential, it therefore mediates more sodium influx than Na_V_1.7. Furthermore, the major ion channels affecting CV are Na_V_1.8 and, to a lesser extent, K_dr_ and Na_V_1.7. It is indeed expected that the major Na_V_ action currents are important in determining CV. Moreover, supernormality is enhanced if Na_V_1.7 channel expression is reduced, because this current is considerably inactivated during the after-depolarization. For instance, during stimulation with two subsequent pulses at short intervals (e.g. 50 ms), the Na_V_1.7 current will be smaller at the time of the second pulse compared to the first.

### Electrical activation thresholds

In this study, we show that alterations in ion channel and pump expression levels are unlikely to underlie the empirically observed differences in electrical thresholds in CM and CMi fibres. The simulations therefore support the suggestion that threshold differences are due to differences in extra-axonal factors such as nerve terminal diameter and fibre depth, with CMi being located deeper in the skin than CM fibres [16]. If this holds true, then discharging the membrane capacitance in CMi nociceptors would require considerably larger transcutaneous currents. In contrast, the current required to activate an axon using intracutaneous stimulation would be expected to be similar for CM and CMi fibres. Our *in vivo* recordings showed that, in the pig, CMi and CM nociceptors have similar rheobase when stimulated intracutaneously, while transcutaneous stimulation of porcine CMi gave a much larger rheobase value than CM. This supports the notion that extra-axonal factors are responsible for the difference in electrical threshold between CM and CMi fibres [16].

### Robustness of the model

In order to assess the influence of parameter changes on the five different stimulation protocols we developed a scoring system. The choice of scoring system is by definition somewhat arbitrary. In an attempt to reduce the bias of the used scoring system, we examined several alternative scoring systems in addition to the three presented in Figure 5. We did not observe any qualitative change in the conclusions presented here. The top candidates, as shown in Figure 5 (transition from CMi to CM: decreased K_dr_ and Na_V_1.8, increased Na_V_1.7, K_M_ and pump), remained the same, albeit with variations in their mutual order.

### Physiological and clinical relevance

Intradermal NGF injections in pig resulted in a dose- and time-dependent ADS reduction that appears to be restricted to CMi nociceptors (Fig. 1B and [13]). Most probably, NGF binds to its high-affinity trkA receptor, and the complex is transported axonally into the somata, where it modifies expression patterns. Consistent with this hypothesis, the NGF-evoked ADS changes observed in our animal studies developed with a delay of ∼5 days. Importantly, NGF is expected to modulate the expression and/or activity of multiple axonal ion channels and pumps, some of which were highlighted by our model as being potentially responsible for the difference of axonal properties between CMi and CM neurons. Specifically, NGF changes the expression levels of voltage-dependent sodium channels (e.g., Na_V_1.7 and Na_V_1.8) and potassium channels (delayed rectifier and M-currents) [25]–[29]. In addition, NGF also alters the activity of the Na^+^/K^+^ -ATPase [30] and together, these changes could have an impact on the resting membrane potential.

### Na_V_1.7 and Na_V_1.8 in pathological pain

A recent modelling study has shown that the relative expression levels of Na_V_1.7 and Na_V_1.8 channels are important in tuning the excitability of nociceptive DRG neurons [31]. The results presented here support this concept. Specifically, we have shown that Na_V_1.7 and Na_V_1.8 act antagonistically on both supernormal excitability and ADS. Supernormality, which has been associated with increased discharge frequency and therefore increased pain perception [7], was strongly reduced when Na_V_1.7 expression was increased. Our simulations suggest that CM fibres have a higher ratio of Na_V_1.7 to Na_V_1.8 mediated currents than CMi fibres and that increasing Na_V_1.7 conductance tends to make CMi fibres more CM like. Gain-of-function mutations in Na_V_1.7 are shown to be responsible for inherited pain syndromes such as erythromelalgia and paroxysmal extreme pain disorder [32]. Patients suffering from these syndromes also display mechanically induced pain. Increasing Na_V_1.7 activity in CMi fibres may render them more similar to CM fibres, potentially supporting an increased sensitivity to mechanically induced pain, like the one observed in these patients.

### Multiple channels lead to multiple hypotheses

It is often argued that models should be kept simple, incorporating as few components as possible. According to such a philosophy, it would be better to use a model with e.g. one K_V_ and one Na_V_ channel, in line with the basic action potential-propagation model formulated by Hodgkin and Huxley [33]. However, the results presented in this study, which are based on a model consisting of several subtypes of K^+^ and Na^+^ channels, point to the opposite. For instance, our simulations predict that two K_V_ channels (K_dr_ and K_M_) have opposing influences during the supernormal phase. This feature is also apparent for two Na_V_ channels (Na_V_1.7 and Na_V_1.8, Figure 4). The model makes predictions that could be tested experimentally, and suggests that specific K_V_ or Na_V_ subtypes may play individual and even opposing roles in regulating nerve fibre excitability. Moreover, Na_V_ channels that are preferentially expressed in nociceptors (e.g. Na_V_1.7, Na_V_1.8) and contribute to action potential generation and conduction are attractive pharmacological targets for pain treatment. The present study additionally shows an unexpected role of K channels (in particular K_dr_) in axonal propagation, a feature potentially relevant in the search for pharmacological targets.

### Natural ion channel-density variation and patient-specific pharmacology

Our correlation analysis showed that, consistent with experimental observations, strong ADS was associated with a strong tendency towards conduction failure and with large supernormal latency shifts (Figure 7). We did not observe a correlation between CV and ADS consistent with other reports [7], although one study published a negative correlation for C-fibres [16]. This observation may be due to natural variations in channel densities across a population of healthy individuals, in line with results from combined computational-electrophysiology studies of the heart [34]. Taking the Na_V_ channels as an example (indicated by solid and dashed lines in Figure 7D), one could speculate that Na_V_1.7, but not Na_V_1.8, is likely to vary within CM and CMi populations. This variation stems from changes in Na_V_1.7 (but not Na_V_1.8) channel density which lead to fibres that follow the trend observed by Weidner et al.[156; namely that high values of CV are associated with weak ADS. An exciting possibility emerging from these ideas is that of ‘personalized medicine’ [34], i.e. that patients could be differentially treated depending on their individual set of C-fibre ion channel densities (as assessed by stimulus protocols such as the ones used in the present study). Assume, for the sake of the argument, that future pharmacology will include both Na_V_1.7 and Na_V_1.8 selective channel blockers, with each drug being associated with a side effect. In selected treatment groups, a patient's drug could then be customized to optimize its efficiency according to model-based insights and results from that patient's microneurographic recordings.

## Summary

In summary, our simulations identified the delayed rectifier potassium channel (K_dr_), the voltage-gated sodium channel Na_V_1.7 and the Na^+^/K^+^-pump as the largest contributors underlying the axonal differences between CM and CMi nociceptors. The *in silico* approach generated hypotheses for the observed shift in nociceptor classes following the application of the hyperalgesic agent nerve growth factor. The identification of Na_V_1.7 in particular is in accordance with a role of this sodium channel in chronic pain conditions. We therefore conclude that *in silico* modelling represents a complementary approach to generate mechanistic hypotheses that can be used to prioritize targets for experimental interventions.
